# Supplementary material for: Decline towards extinction of Mexico's vaquita porpoise (Phocoena sinus)
Source: R Soc Open Sci. 2019 Jul 31;6(7):190598. doi: 10.1098/rsos.190598 (PMC6689580; doi:10.1098/rsos.190598)

Supplementary text accompanying Jaramillo-Legorreta, "Decline towards extinction of Mexico's vaquita porpoise (Phocoena sinus)"

Appendix S1. Further details on geospatial model

Table S1: Prior distributions for the geospatial model

Parameter	Prior distribution
μ_{2011}	Uniform(-10, 10)
μ_{2012}	Uniform(-10, 10)
μ_{2013}	Uniform(-10, 10)
μ_{2014}	Uniform(-10, 10)
μ_{2015}	Uniform(-10, 10)
μ_{2016}	Uniform(-10, 10)
μ_{2017}	Uniform(-10, 10)
μ_{2018}	Uniform(-10, 10)
σ_{ε}^2	Uniform(0, 10)
σ_z^2	Uniform(0, 10)
ρ	Uniform(0, 500)

Figure S1. Marginal predictive p-values for the geospatial model. The p-value is calculated as the proportion of data values simulated from the posterior distribution of model parameters that are greater than or equal to the observed data value. Values close to 1 or 0 indicate that the model generally over- or under-predicts the corresponding observed data value. Only one site has values outside the range 0.05-0.95, in two years (highlighted with thick red circles). Size of circles indicates amount of sampling effort in the corresponding year; those with Xs had no effort.

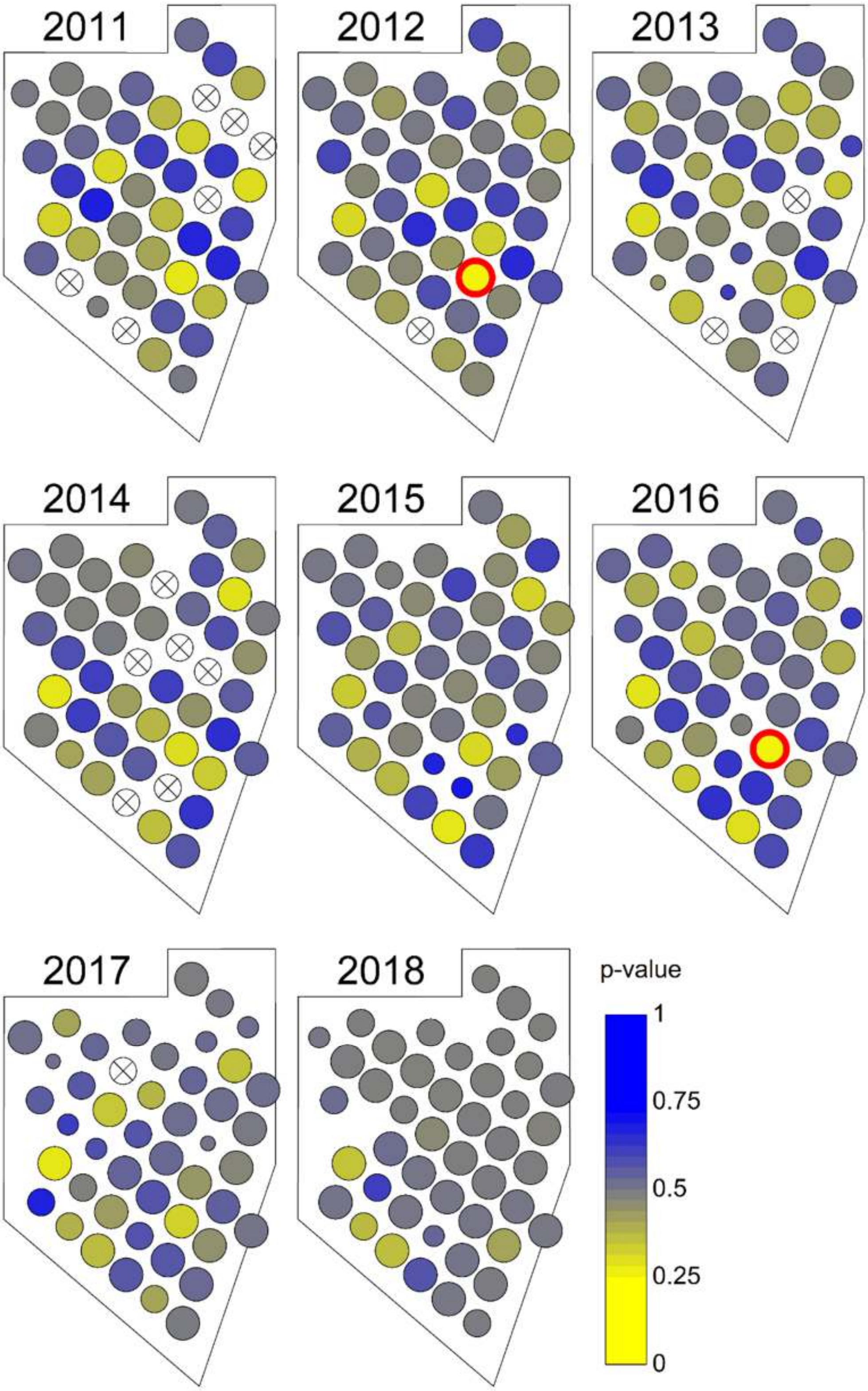

Appendix S2. Non-spatial mixture model

In previous papers [1, 3] a non-spatial mixture model was used in addition to the geospatial model to make inferences about acoustic trends. The model makes different assumptions, and combining inferences from both models was considered to increase robustness of the results. However, as shown below, with the longer time series now available, the model no longer fits the data well, and so was not used in the main paper for inference. We present model results here for comparison.

Full model specifications for this model are in Jaramillo-Legorreta et al. [1]; code and data to fit the model are included as part of these Supplementary Materials. Briefly, the non-spatial mixture model probabilistically assigns individual C-POD locations to one of three strata (low, medium, or high click rate) and provides modeled estimates of the mean daily click rate for each stratum. A sampling site is permanently assigned to the same stratum for all years (which is justified based on spatial stability of the data), but the stratum rates are estimated independently for each year. The purpose of stratification is to statistically account for much of the inter-site variance in the number of clicks recorded. Annual click counts at each site are treated as negative binomial random variables with the expectation given by the product of location-specific effort and stratum-specific click rates and over dispersion. Inference is based on annual differences in the mean of the modeled click rate estimates for the 46 sites.

Like the geospatial model, the mixture model was formulated in a Bayesian framework using non-informative prior distributions on model parameters. Inference was via Markov chain Monte Carlo, implemented in OpenBUGS via and R script. Initialization procedures, burn-in, sample lengths and thinning were the same as for the geospatial model. Geweke's and Heidelberger and Welch's criteria were used to diagnose convergence, and marginal predictive *p*-values calculated to assess goodness-of-fit.

Estimated annual changes (Table S2) were generally similar to those from the geospatial model, and very similar for the later years where there were fewer missing data. The overall geometric mean population change, averaging over all years of monitoring, was nearly identical (0.54 for the misture model vs 0.53 for the geospatial model).

Unlike the geospatial model, however, the posterior predictive checks revealed significant problems with goodness-of-fit: many of the marginal *p*-values were close to 0 or 1, particularly in the later years, indicating that the model over- or under-predicted acoustic activity at many sites (Figure S2). This is likely due to violation of the model assumption that sites are consistently in the same stratum of acoustic activity. Given the range contraction that has accompanied the decline in acoustic activity over the years, some sites that may previously have been in the medium or high activity stratum now have no acoustic detections. Despite this lack of fit at the site by year level, predictions at the year level were reasonable: marginal *p*-values calculated for average annual click count were all adequately close to 0.5 (values for 2011-2018 were 0.49, 0.51, 0.49, 0.47, 0.45, 0.48, 0.43 and 0.64, respectively).

Table S2. Estimated per-year change (λ) in acoustic activity from the non-spatial mixture model to acoustic monitoring data, before and after incorporation of the additional sightings of vaquita in 2017 and 2018. Quantities are posterior means with 95% posterior credible intervals in brackets.

	Before incorporation of 2017 and 2018 sightings	After incorporation of 2017 and 2018 sightings	Probability Declining	Probability declining > 20%/year
2011-12	1.01 (0.42 – 2.24)	1.01 (0.40 – 2.23)	60.5%	37.9%
2012-13	0.76 (0.25 – 1.80)	0.76 (0.24 – 1.81)	80.3%	63.8%
2013-14	0.52 (0.21 – 0.96)	0.53 (0.21 – 0.97)	98.0%	91.8%
2014-15	0.64 (0.42 – 0.93)	0.63 (0.42 – 0.89)	99.2%	91.7%
2015-16	0.58 (0.36 – 0.94)	0.60 (0.37 – 0.96)	97.8%	91.6%
2016-17	0.44 (0.19 – 1.00)	0.44 (0.18 – 1.02)	97.2%	94.0%
2017-18	0.37 (0.11 – 0.92)	0.48 (0.15 – 1.12)	95.8%	90.4%
Geometric mean per-year change	0.54 (0.48 – 0.62)	0.56 (0.50 – 0.63)	≈100%	≈100%

Figure S2. Marginal predictive p-values for the non-spatial mixture model. The p-value is calculated as the proportion of data values simulated from the posterior distribution of model parameters that are greater than or equal to the observed data value. Values close to 1 or 0 indicate that the model generally over- or under-predicts the corresponding observed data value. Many sites have values outside the range 0.05-0.95. Size of circles indicates amount of sampling effort in the corresponding year; those with Xs had no effort.

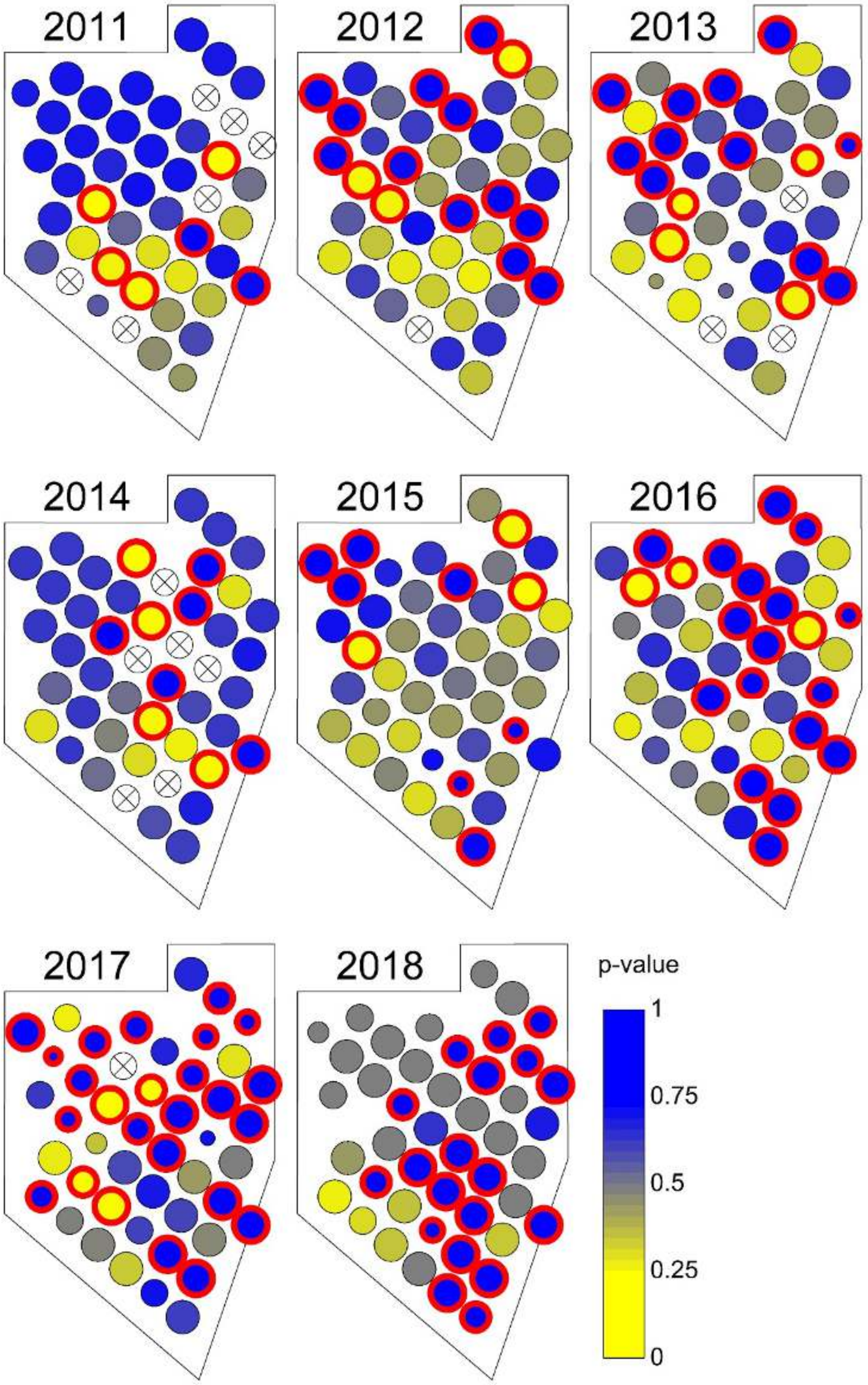

Supplement: Model descriptions [file rsos190598supp2.pdf]
